# Non-uniform failure and differential pressure relief technology of roadway under irregular goafs in deep close-distance coal seams

**DOI:** 10.1038/s41598-023-45857-y

**Published:** 2023-10-28

**Authors:** Shuaifeng Yin, Xinjian Zheng, En Wang, Qingtao Kang, Xiaoming Zhang

**Affiliations:** 1 School of Mine Safety, North China Institute of Science and Technology, Langfang, 065201 China; 2 School of Energy and Mining Engineering, China University of Mining & Technology-Beijing, Beijing, 100083 China

**Keywords:** Civil engineering, Engineering

## Abstract

In response to the control problems of large-deformation roadways in close-distance coal seams, taking a typical roadway under irregular goafs and residual coal pillar in deep close-distance coal seams as the background, the characteristics of mine pressure and key difficulties in surrounding rock control of roadway are explored and an improvement strategy for controlling surrounding rock is proposed. The stress expression in roadway floor under the influence of residual coal pillar is derived by theoretical calculation. The peak lines of deviatoric stress and vertical stress in roadway after the mining of the upper coal seam are obtained by numerical simulation. The roadway is divided into two key zones: ordinary zone and disturbance zone by residual coal pillar, and the disturbance range of roadway below residual coal pillar is determined to be 44.60 m. It reveals the differential, asymmetric, and non-uniform distortion and failure laws of roadway at different positions under irregular goafs and residual coal pillar. The differential control technology named asymmetric support in ordinary zone of roadway and combined support and drilling pressure relief in disturbance zone below residual coal pillar is proposed. The feasibility of differential pressure relief and control technology has been verified through on-site engineering test, which ensures the safety and stability of roadway and provides technical references for surrounding rock control in similar deep and complex roadways.

## Introduction

With the gradual depletion of shallow coal resources in China, the extension of mines to the deep has become an inevitable trend in coal resource development^1–3^. After entering the deep mining stage, it often causes many problems such as increased ground stress, complex geological condition, and intensified rock damage and fracture^4,5^. For close-distance coal seams mining, due to the disturbance of the upper coal seam mining and residual coal pillars, its mine pressure in roadway is significantly abnormal, which will cause more prominent safety issues. Therefore, determining the failure mechanism and reasonable pressure relief and control technologies for roadway surrounding rock is of great significance for ensuring the safe and efficient mining of the lower coal seam in highly productive coal mines^6^.

Scholars have conducted extensive research on the failure mechanism and control technology of roadway surrounding rock in close-distance coal seams by theoretical calculation, numerical simulation, similarity simulation, and engineering analogy. Shang et al.^7^ used on-site measurement and numerical simulation to study the stress distribution and transfer laws of residual coal pillar floor in close-distance coal seams, revealing the failure mechanism of roadway under irregular goafs. Ning et al.^8,9^ analyzed the fracture laws of overlying rock under close-distance coal seams, further revealing the evolution of upper strata fractures and surface subsidence caused by intense-mining panel extraction. Based on close-distance coal seams as background, Zhang et al.^10^ calculated the failure depth of underlying rock strata in the goaf, revealed the instability and fragmentation mechanisms of gob-side entry retaining below the goaf, and determined the basic principles for controlling the stability of roadway. Sakhno & Sakhno^11^ revealed the mechanisms of floor heave failure and instability of surrounding rock in deep soft roadway, and proposed corresponding grouting modification technology. Tan et al.^12^ divided the goaf floor into four failure zones, and proposed a new on-site survey technology that can identify the loose and damaged zone of surrounding rock. Shang et al.^13^ studied the characteristics of overlying rock fracture and gas migration after the mining in close-distance coal seams. Wang et al.^14,15^ analyzed the spatiotemporal distribution and dynamic migration laws of toxic gases in the goaf behind the panel during the mining process of close-distance coal seams, and formulated corresponding preventive measures. Zhang et al.^16,17^ elucidated the characteristics of the impact of residual coal pillar in close-distance coal seams on mine pressure, obtained the failure depth and mechanism in goaf floor, and revealed the evolution laws of stress before and after pressure relief in panel of close-distance coal seams. Lu and Ni^18^ constructed a mechanical model of roadway floor rock in close-distance coal seams, and obtained reasonable support parameters, grouting material ratio, and grouting pressure for roadway surrounding rock. Aghababaei et al.^19^ used a rock engineering system to study the floor deformation mechanisms at longwall face in a coal mine and conducted in-depth risk analysis and prediction.

As of now, there are few reports on differential pressure relief and control technology for roadways under irregular goafs in close-distance coal seams. In view of this, the study takes a coal roadway under irregular goafs in deep close-distance coal seams as engineering background to elucidate the key difficulties and improvement strategy for roadway surrounding rock control. According to the distribution laws of stress field, the roadway is divided into two key zones, revealing the asymmetric and non-uniform instability characteristics of roadway under irregular goafs and residual coal pillar. We propose a differential pressure relief and control technology for coal roadway in different zones, and verify the rationality of above results by on-site test, which provides technical references for the control of deep complex and large-deformation roadways.

## Engineering overview of roadway in close-distance coal seams

This section mainly introduces the engineering geological overview, mine pressure characteristic, and key difficulties in surrounding rock control of test roadway, and proposes improvement strategies for roadway control.

### Engineering geological overview

**Figure 1:**
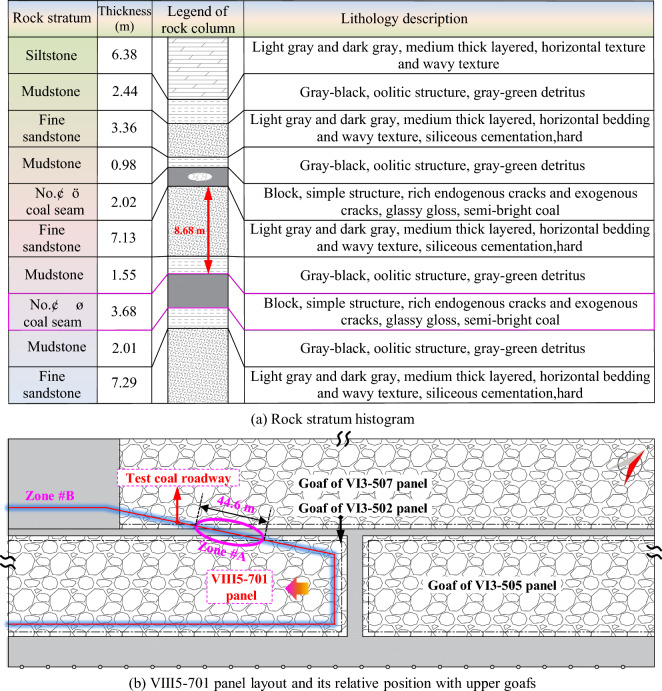
Rock strata histogram and layout of VIII5-701 panel.

The experimental mine mainly mines No. 1, 3, 5, VI, and VIII coal seams, and adopts a downward mining method for recovery. At present, the panels in No. VI coal seam have been mined out, and it is about to mine the lower panels in No. VIII coal seam. The No. VIII coal seam has a thickness of 3.68 m and an inclination angle of 6°, belonging to a nearly-horizontal coal seam. The distance between the upper and the lower coal seams is 8.68 m, which belongs to close-distance coal seams. The VIII5-701 panel in No. VIII coal seam is the first mining panel, with an average burial depth of 860 m, belonging to a deep coal mine. The top of this panel is irregular goafs of VI3-502, VI3-505, and VI3-507 panels and residual coal pillar. The test roadway is a mining roadway in VIII5-701 panel, which passes through irregular goafs above and residual coal pillar. The cross-section of the rectangular roadway is 5.0 × 3.0 m, which is arranged along the roof in No. VIII coal seam. The rock column and layout of VIII5-701 panel are shown in Fig. 1.

### Mine pressure characteristics

**Figure 2:**
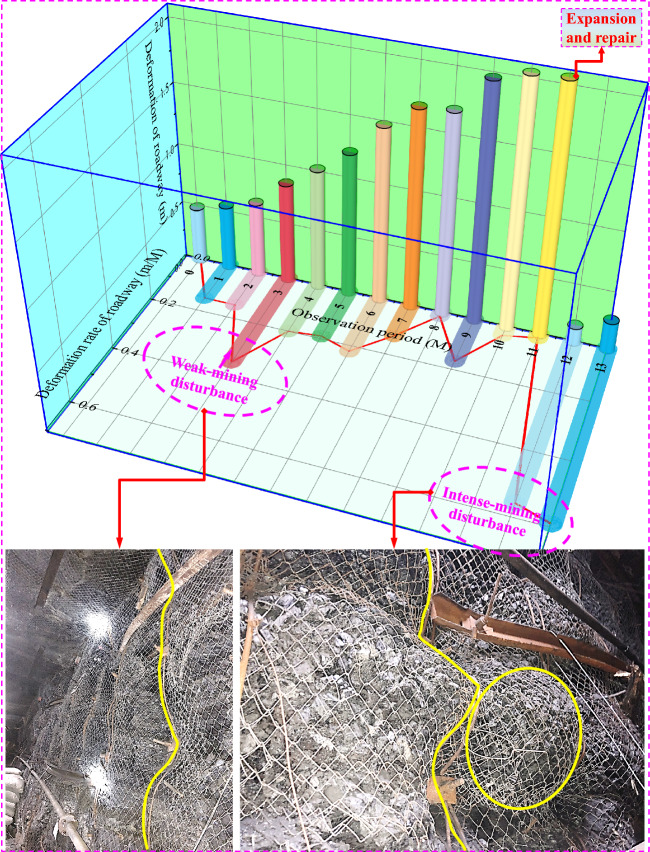
Deformation and failure of adjacent roadway.

Due to the VIII 5-701 panel is the first mining zone in No. VIII coal seam, there is no experience available for reference regarding the characteristics of mining pressure behavior and roadway support design. In view of this, the deformation and failure processes and characteristics of roadway surrounding rock during the mining in adjacent panels are collected and analyzed, as shown in Fig. 2. Due to the roadway is located in a deep coal seam, under the deep and complex conditions of high ground stress, the roadway undergoes continuous deformation, and deformation rate gradually accelerates by the mining disturbance. The maximum deformation of roadway after mining disturbance exceeds 2.0 m, and the basic functions of roadway cannot be continued. In the long run, regular expansion and repair are needed to maintain its basic operation. The deformation and damage of roadway mainly manifest as the bending and sinking in the roof, severe heave in two ribs, failure of anchor cables and bolts, and damage to metal mesh. Due to the roadway is in a complex condition under deep irregular goafs and residual coal pillar, its control difficulty is significantly greater than that of roadways in ordinary engineering.

### Key control difficulties

**Figure 3:**
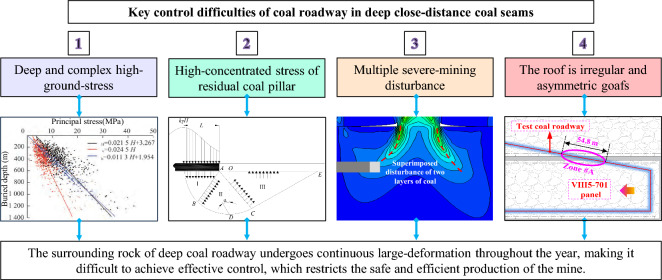
Key difficulties of roadway surrounding-rock control in close-distance coal seams.

Based on the engineering conditions of test roadway, combined with the mine pressure of adjacent roadway surrounding rock, the key difficulties (Fig. 3) in controlling roadway surrounding rock are analyzed as follows:

1. Deep and complex high-ground-stress^20,21^. The panel is buried at a depth of 860 m, which belongs to high ground stress of deep coal mine. The mechanical environment, organizational structure, and basic behavior in deep surrounding rock make the roadway exhibit intense mine pressure characteristics such as large deformation, fast convergence rate, long continuous-deformation period, and damage to support system.
2. High-concentrated stress of residual coal pillar in close-distance coal seams. Some zones of test roadway are located below residual coal pillar in the upper coal seam. Due to the accumulation of a large amount of distortion energy in residual coal pillar, the roadway stress within a certain range below residual coal pillar will be highly concentrated, making it difficult to control its surrounding rock.
3. Multiple severe-mining disturbance. The mining of the upper coal seam panels will cause certain disturbance and damage to floor surrounding rock, and at the same time, the mining of the lower coal seam panel will have a more severe impact on roadway. Multiple severe-mining effects of two layers of coal seam will lead to a larger range and greater degree of distortion and damage to coal roadway.
4. The roadway roof is irregular and asymmetric goafs. Due to the irregular and asymmetric goafs and residual coal pillar above the roadway, it will experience non-uniform and differential failure characteristics under the complex engineering condition, so it is necessary to design a differential pressure relief and control technology.

### Improvement strategies for roadway surrounding-rock control

Based on the key difficulties in controlling roadway surrounding rock, the improvement strategies for achieving stability control of roadway under irregular goafs in deep close-distance coal seams are as follows: (1) In terms of roadway zoning, the coal roadway is divided into two key zones: ordinary zone and disturbance zone affected by residual coal pillar. (2) In terms of roadway control: on the one hand, it is necessary to strengthen support for shallow surrounding rock of coal roadway; and on the other hand, it is necessary to implement drilling pressure relief technology for roadway under residual coal pillar to improve its stress environment^22–24^.

## Non-uniform instability characteristics of roadway in close-distance coal seams

Based on overlying rock structure characteristics of roadway under irregular goafs in close-distance coal seams, the stress expression at any point below residual coal pillar is derived. We study the evolution laws of plastic failure field and stress field of surrounding rock above roadway, revealing the differential and non-uniform failure characteristics of roadway, thereby guiding the stability control of roadway surrounding rock.

### The overlying rock structure and failure characteristics of roadway in close-distance coal seams

**Figure 4:**
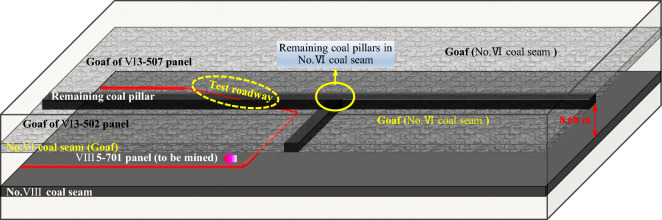
Spatial structure of roadway surrounding rock in close-distance coal seams.

Based on engineering overview of VIII5-701 panel, the overlying rock structure of test roadway is shown in Fig. 4. The roadway passes through the VI3-507 goaf, a 8.0 m-width coal pillar, and the VI3-502 goaf from the open-off cut. From this, it can be seen that the upper of test roadway is irregular and asymmetric goafs and residual coal pillar, which will cause asymmetric and non-uniform damage under the spatial structure.

**Figure 5:**
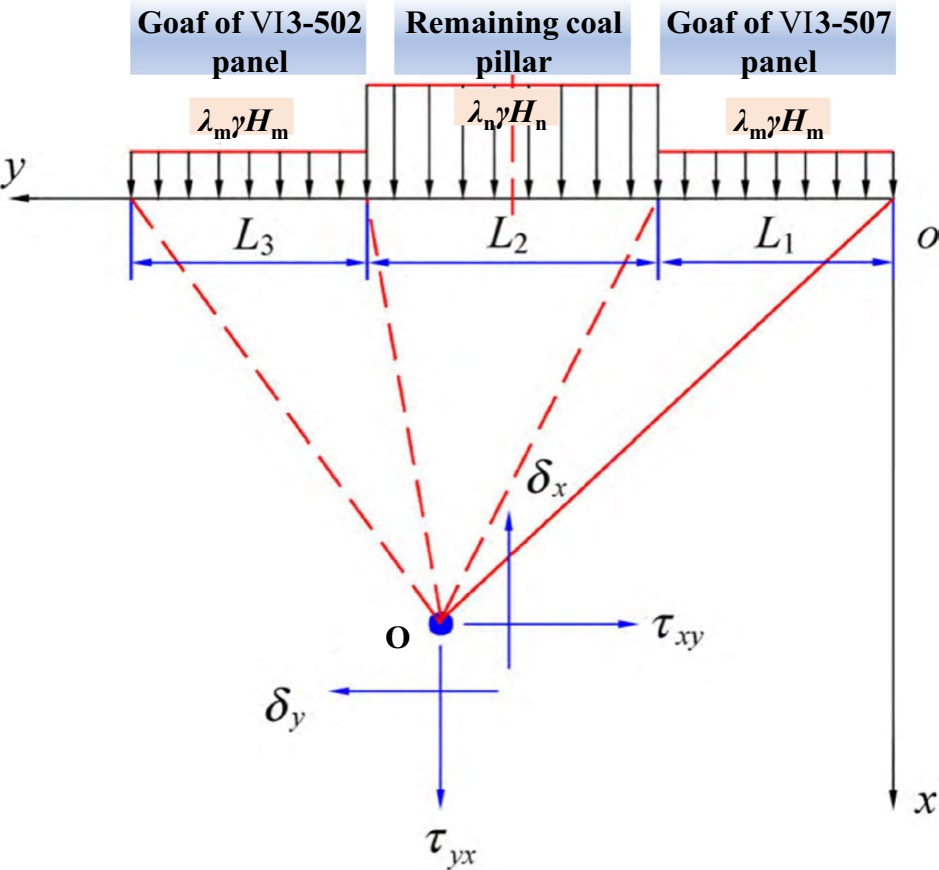
Force analysis on the floor within the influence of residual coal pillar.

In order to further analyze the stress distribution of roadway surrounding rock under residual coal pillar, a cross-section is made along the excavation direction of roadway, and a stress model of any point on the floor within the influence range of residual coal pillar is established based on the half plane theory^25,26^, as shown in Fig. 5.

Where, *γ* and *H*_m_ are the average bulk density and average burial depth of overlying rock, respectively.

*λ*_m_ is stress concentration coefficient within the pressure relief protection range of the upper goaf.

*λ*_n_ and *H*_n_ are stress concentration coefficient and average burial depth of residual coal pillar, respectively.

O is any point within the floor of coal and rock mass.

*δ*_x_, *δ*_y_, and *τ*_xy_ represent the horizontal stress, vertical stress, and shear stress on point O, respectively.

Based on existing results, it can be inferred that due to the extremely small bearing capacity of the gangue at the edge of residual coal pillar, it can be approximately assumed to be 0. As it moves away from the edge of residual coal pillar, its bearing capacity gradually increases with the increase of compaction degree, and finally recovers to original rock stress state at a position 0.12–0.30 times the burial depth of residual coal pillar. For the convenience of calculation, the bearing pressure on the floor within the pressure relief protection range is averaged, and the average is *λ*_m_*γH*_m_, and the average stress of residual coal pillar on the floor is *λ*_n_*γH*_n_.

The stress analysis of any point O in the floor of coal and rock mass is as follows. Firstly, taking the influence range (width *L*_1_) of pressure relief protection in VI3-507 goaf as object, any small unit length d*η* is intercepted within its corresponding width influence range, and the uniformly distributed load *q*_1_(*η*) within this small unit is:

1 $$q_{1} (\eta ) = \lambda_{{\text{m}}} \gamma H_{{\text{m}}} d\eta$$

The stress effect of uniformly distributed load *q*_1_(*η*) on any point O in the floor is as follows:

2 $$\left\{ \begin{gathered} {\text{d}}\delta_{x}^{1} = - \frac{{2\lambda_{m} \gamma H_{m} {\text{d}}\eta }}{\pi }\frac{{x^{3} }}{{\left[ {x^{2} + (y - \eta )^{2} } \right]^{2} }} \hfill \\ {\text{d}}\delta_{y}^{1} = - \frac{{2\lambda_{m} \gamma H_{m} {\text{d}}\eta }}{\pi }\frac{{x(y - \eta )^{2} }}{{\left[ {x^{2} + (y - \eta )^{2} } \right]^{2} }} \hfill \\ {\text{d}}\tau_{xy}^{1} = - \frac{{2\lambda_{m} \gamma H_{m} {\text{d}}\eta }}{\pi }\frac{{x^{2} (y - \eta )}}{{\left[ {x^{2} + (y - \eta )^{2} } \right]^{2} }} \hfill \\ \end{gathered} \right.$$

where, $\delta_{x}^{1}$, $\delta_{y}^{1}$, and $\tau_{xy}^{1}$ are the horizontal stress, vertical stress, and shear stress at any point O under uniformly distributed load *q*_1_(*η*), respectively, MPa; *x* and *y* are the horizontal and vertical coordinates, respectively, m, relative to any point O in Fig. 5.

3 $$\left\{ \begin{gathered} \delta_{x}^{1} = - \frac{{2\lambda_{m} \gamma H_{m} }}{\pi }\int_{0}^{{L_{1} }} {\frac{{x^{3} }}{{\left[ {x^{2} + (y - \eta )^{2} } \right]^{2} }}{\text{d}}\eta } \hfill \\ \delta_{y}^{1} = - \frac{{2\lambda_{m} \gamma H_{m} }}{\pi }\int_{0}^{{L_{1} }} {\frac{{x(y - \eta )^{2} }}{{\left[ {x^{2} + (y - \eta )^{2} } \right]^{2} }}{\text{d}}\eta } \hfill \\ \tau_{xy}^{1} = - \frac{{2\lambda_{m} \gamma H_{m} }}{\pi }\int_{0}^{{L_{1} }} {\frac{{x^{2} (y - \eta )}}{{\left[ {x^{2} + (y - \eta )^{2} } \right]^{2} }}{\text{d}}\eta } \hfill \\ \end{gathered} \right.$$

Similarly, the surrounding rock within the influence range of residual coal pillar (width *L*_2_) and VI3-502 goaf (width *L*_3_) is taken as object, and any small unit length d*η* is intercepted within its corresponding width influence range. Based on Eqs. (2) and (3), the corresponding stress impact on any point O in the floor can be obtained. Finally, based on the principle of stress superposition, the final force magnitude of any point O in the floor under the influence of pressure relief protection in VI3-507 goaf (width *L*_1_) and VI3-502 goaf (width *L*_3_), as well as the influence of residual coal pillar (width *L*_2_), can be derived, as shown in Eq. (4):

4 $$\left\{ \begin{gathered} \delta_{x} = \delta_{x}^{1} + \delta_{x}^{2} + \delta_{x}^{3} \hfill \\ \delta_{y} = \delta_{y}^{1} + \delta_{y}^{2} + \delta_{y}^{3} \hfill \\ \tau_{xy} = \tau_{xy}^{1} + \tau_{xy}^{2} + \tau_{xy}^{3} \hfill \\ \end{gathered} \right.$$

where, $\delta_{x}^{i}$, $\delta_{y}^{i}$, and $\tau_{xy}^{i}$ are the stresses (horizontal stress, vertical stress, shear stress) of width *L*_*i*_ at any point O in the floor, respectively, MPa; And the values of *i* are 1, 2, and 3, respectively.

Based on the engineering conditions, the horizontal stress, vertical stress, and shear stress at any point O in the floor under the influence of residual coal pillar can be calculated, thereby analyzing and calculating the damage range under the disturbance of overlying panel mining, and guiding the safe mining in No. VIII coal seam and support design of roadway surrounding rock.

### Evolution laws of roadway stress field under the influence of intense mining

#### Numerical model

Based on engineering conditions of test roadway, a large-scale three-dimensional numerical model is constructed using FLAC3D software that is consistent with its actual on-site engineering. The advancing direction of the panel is the X-axis (taken as 200 m), the tilting direction of the panel is the Y-axis (taken as 180 m), and the vertical direction is the Z-axis (taken as 80 m). The top boundary of the model is stress constrained, with zero velocity in the X direction of the left and right boundaries, zero velocity in the Y direction of the front and rear boundaries, and zero velocity in the X, Y, and Z directions of the bottom boundary. The lateral pressure coefficient is 1.2. According to the burial depth of 860 m for the coal roadway in VIII5-701 panel, the load applied to the top of the model is 21.50 MPa. The Mohr–Coulomb model is used as the constitutive model for the deformation and failure of roadway surrounding rock. Since the presence of joints and cracks in coal and rock mass in the coal mine, and differences from laboratory standard samples, the mechanical parameters of coal and rock mass used for numerical simulation are obtained by processing the parameters measured in the laboratory using the Hoek–Brown criterion^27–29^.

#### No goaf on the upper rock

**Figure 6:**
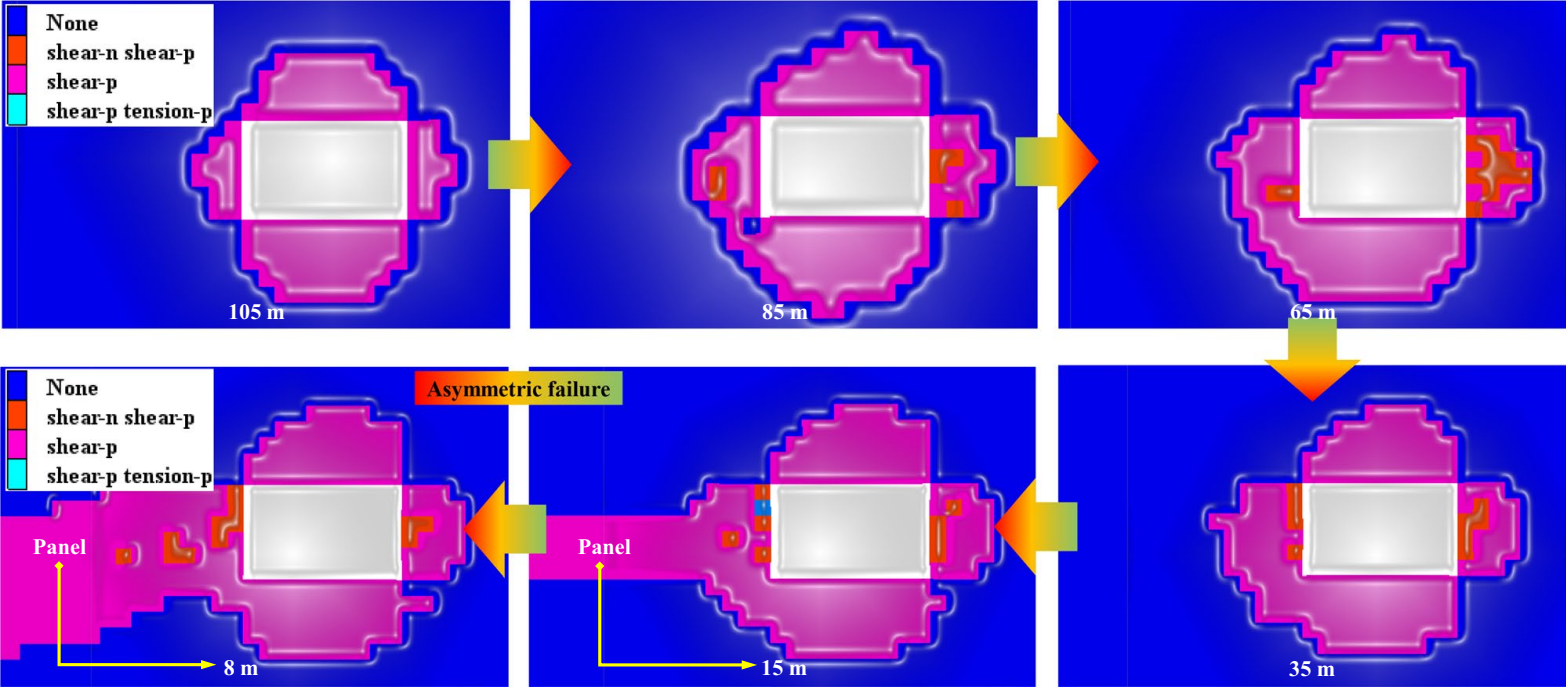
Evolution process of plastic zone for roadway and panel position when there is no overlying goaf.

When there is no goaf on the roof of the panel, the plastic failure process of surrounding rock from excavation to mining in test roadway is shown in Fig. 6. After the excavation of coal roadway, the shallow surrounding rock undergoes a certain degree of plasticization failure, exhibiting approximately symmetrical deformation and failure characteristics. As coal roadway is affected by the mining disturbance of the panel, the damage range of surrounding rock near the mining rib gradually deepens. As the distance from the mining panel gets closer, the damage range of roadway surrounding rock reaches its maximum, and the maximum range of damage can extend to the deep coal of mining rib. Overall, the roadway surrounding rock mainly undergoes shear and tensile failure.

#### There are goafs on the upper rock

**Figure 7:**
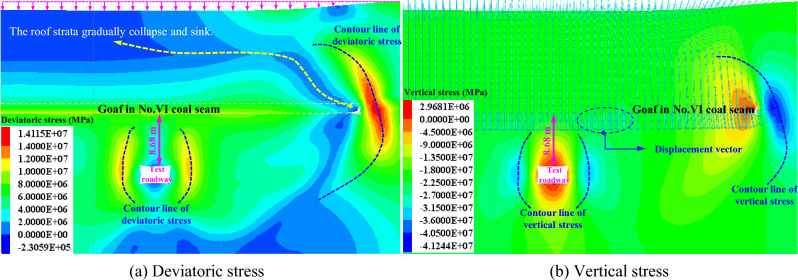
Stress distribution of roadway surrounding rock after overlying panel is mined out.

The vertical stress^30^ is a classic index for analyzing engineering problems in coal mine. The deviatoric stress^31^ can reflect the essential characteristic of rock deformation and failure, and plays an important role in analyzing the instability characteristics of roadway. When there is a goaf at 8.68 m above the coal roadway, the distribution of deviatoric stress and vertical stress in roadway surrounding rock is shown in Fig. 7. Because the upper panels have been mined out, causing its roof to collapse and sink, the gangue in the goaf gradually compacts over time and carries and transmits the load on overlying roof. Due to the influence of mining disturbance on the upper panels, the stress in roadway surrounding rock is redistributed. The high deviatoric stress peak zone and vertical stress concentration zone are formed at the edge of the goaf, forming an upward and downward curved shape, and wrapping shape on both ribs of roadway. The highly concentrated stress will cause severe deformation and failure of roadway surrounding rock.

**Figure 8:**
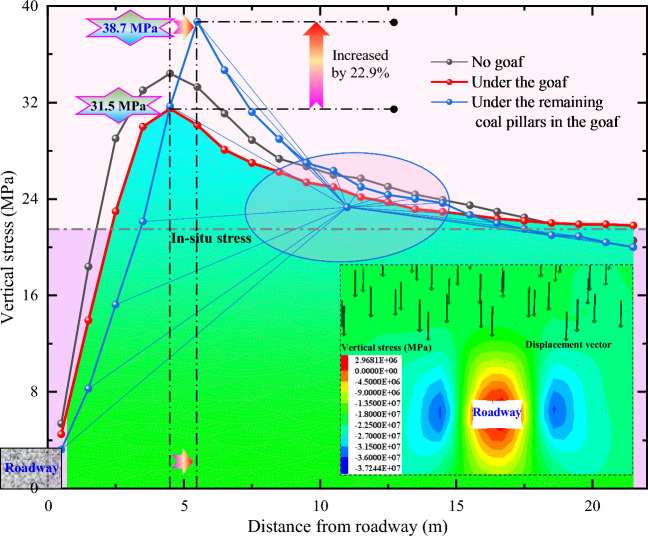
Stress evolution of roadway under different surrounding rock conditions.

In order to further analyze the stress distribution laws of roadway surrounding rock in different engineering conditions, the stress distribution curves of roadway under three conditions (no goaf, under the goaf, and under residual coal pillar) are obtained. The results are shown in Fig. 8. Overall, the roadway stress located below the goaf is the smallest, located at the bottom of all curves, with a peak stress of only 31.50 MPa. When there is no goaf on the roof, the roadway stress is slightly greater than that with goaf, with a peak stress of 34.41 MPa. When the roadway is located below residual coal pillar, the peak stress position is farthest from the roadway, and the maximum stress peak can reach 38.70 MPa, with a stress concentration coefficient of 1.80. Compared with the peak stress of roadway below the goaf, the peak stress increases by 22.9%. From this, it can be seen that the stress in roadway surrounding rock below residual coal pillar is the most concentrated, and it is also the most likely to induce deformation and failure. In view of this, the control difficulty of roadway surrounding rock under residual coal pillar is the greatest, and it is necessary to propose targeted pressure relief and control technology that is different from ordinary roadway.

### Differential and non-uniform characteristics of failure in roadway surrounding rock

**Figure 9:**
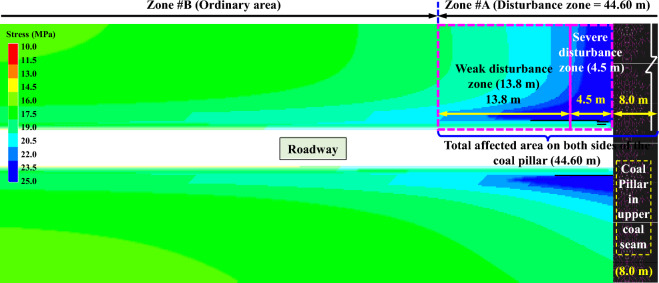
Non-uniform distribution of stress at different positions of roadway below residual coal pillar.

In order to explore the evolution laws of surrounding rock stress during the transition from goaf to residual coal pillar in roadway, a top view of stress along the axial direction of roadway is obtained, as shown in Fig. 9. Due to the influence of high-concentrated stress in residual coal pillar, the stress within a certain range from below residual coal pillar to below the goaf exhibits typical differential and non-uniform characteristics. The stress concentration below residual coal pillar is high, and when transitioning towards the goaf direction, the stress gradually decreases, ultimately tending to a uniform distribution pattern. According to the simulation results in Fig. 9, the impact of residual coal pillar in the upper goaf on the lower roadway mainly includes three parts: (1) the zone directly below the coal pillar, with the width of 8.0 m. (2) Severe disturbance zone, with a unilateral distance of 4.5 m and a total distance of 9.0 m on both sides. (3) Weak disturbance zone (13.80 m), the total distance affected by both sides can reach 27.60 m. Therefore, based on stress distribution of surrounding rock along the axial direction of roadway, it can be divided into two zones, namely ordinary zone (Zone #B) and disturbance zone (Zone #A = 44.60 m) affected by residual coal pillar. In summary, due to the high concentrated stress of residual coal pillar in No.VI coal seam, it has been determined that the total disturbance distance of residual coal pillar on the lower roadway is 44.60 m, and the roadway exhibits typical differential and non-uniform failure characteristics.

**Figure 10:**
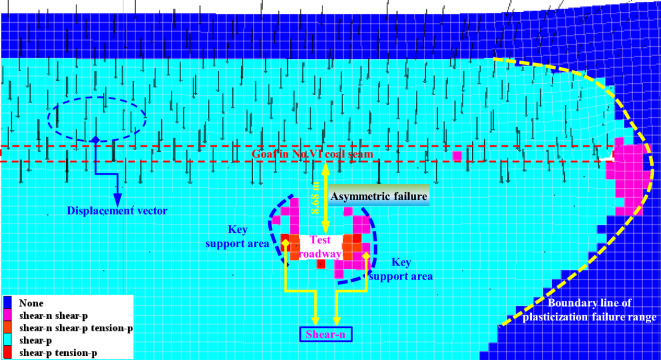
Plastic zone distribution of roadway surrounding rock after overlying panel is mined out.

The plasticization failure range of roadway surrounding rock after the mining of the upper panels in close-distance coal seams is shown in Fig. 10. There is an approximately arc-shaped distribution of plasticizing boundary line along the front of coal wall, which penetrates into a certain range of the top and bottom of the goaf. Due to the fact that the roadway is located within the plasticizing disturbance range of the goafs in the upper coal seam and also within the pressure relief zone after the mining of the upper panels, the roadway surrounding rock is to some extent in pressure relief zone, which is conducive to maintaining the stability of coal roadway. Furthermore, there are obvious shear failure zones at left shoulder and right bottom corners of roadway, exhibiting typical asymmetric characteristic, which are key support areas for roadway surrounding rock. Therefore, inclined anchor cables need to be adopted to strengthen support in roadway support design and ensure that shallow surrounding rock is in a pre-tensioned and stable state.

**Figure 11:**
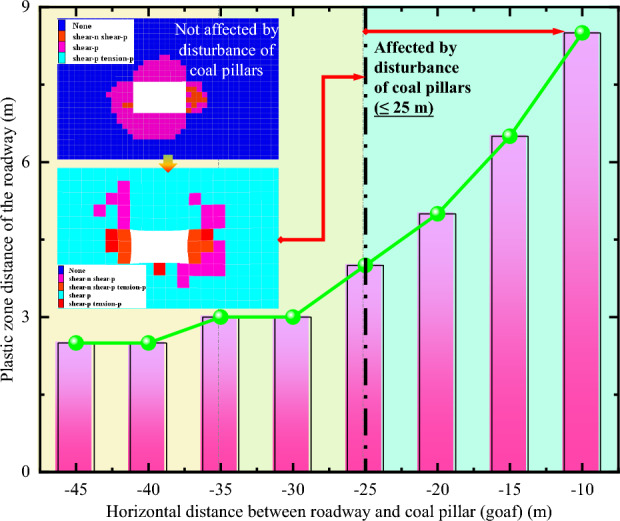
Non-uniform failure process of surrounding rock in different positions of roadway.

In order to further compare the plastic failure status and evolution laws of surrounding rock in entire process of roadway from not being affected by the mining to being affected, the changes of plastic zone in roadway surrounding rock at different positions from the panel are extracted, as shown in Fig. 11. When the distance between roadway and the panel decreases to 25 m, it is in a significant impact zone. The range of plasticization failure of roadway surrounding rock significantly increases, that is, the range of severe disturbance of the panel is approximately 25 m. As the distance from the panel gets closer, the range of plasticization failure in roadway continues to expand. When the distance between the panel and roadway is 10 m, the maximum value of plastic zone can reach 8.5 m, and it exhibits significant differential and asymmetric failure characteristics. Therefore, the closer the location to the mining panel, the greater the difficulty in controlling roadway surrounding rock.

In summary, due to the mining influence of close-distance coal seams and stress concentration of residual coal pillar, the roadway surrounding rock under irregular goafs and residual coal pillar exhibits typical differential, asymmetric, and non-uniform failure patterns. And the roadway is divided into two zones, it is necessary to develop differential pressure relief and control technology for each zone of roadway.

## Differential pressure relief and control technology for roadway in close-distance coal seams

Based on the contour lines of deviatoric stress and vertical stress of roadway surrounding rock after being affected by the mining of close-distance coal seams, combined with the differential, asymmetric, and non-uniform failure patterns of roadway under irregular goafs and residual coal pillar, this section proposes that inclined anchor cables should adopt asymmetric differential arrangement and pass through the contour line of deviatoric stress in two ribs of roadway surrounding rock, so that the end anchor point is located in deep and stable rock mass. By developing differential pressure relief and control technology for two different zones of roadway, we aim to ensure the safety and stability of roadway surrounding rock.

### Asymmetric support technology and parameters

**Figure 12:**
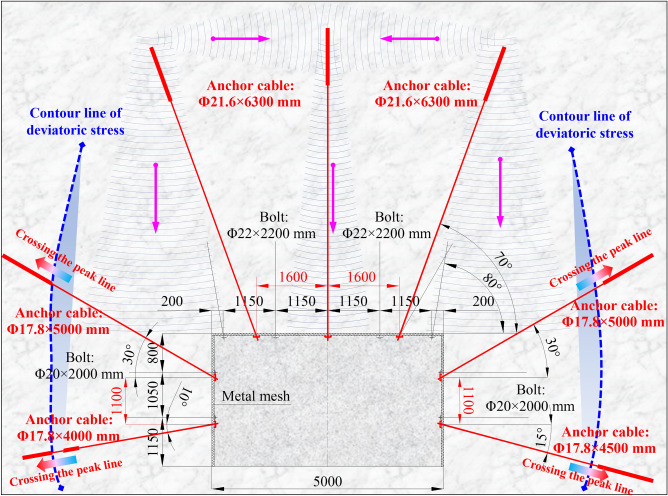
Support technology and key parameters of coal roadway (Unit: mm).

1. Support technology and parameters in roadway roof: The bolt specification in roadway roof is Φ22 × 2400 mm, and the spacing and row is 1150 × 1000 mm. Three anchor cables with a specification of Φ21.6 × 6300 mm are arranged on roadway roof, with the spacing and row is 1600 × 3200 mm. The left and right anchor cables are arranged in a 20° inclined manner towards both ribs, and three anchor cables are connected to the W-steel-strip to form a large-scale prestressed anchoring and bearing structure^32^. The pre-tightening force of anchor cable is not less than 150 kN.
2. Support technology and parameters in roadway ribs: The bolt specification in roadway ribs is Φ20 × 2000 mm, with the spacing and row is 1050 × 1000 mm. Due to the large plastic failure in left shoulder and right floor corners of roadway, the anchor cables in both ribs are arranged in an asymmetric inclined manner. The specification of the upper anchor cable on both ribs is Φ17.8 × 5000 mm, arranged with an upward inclination of 30°. The specification of anchor cable on the lower side of left rib is Φ17.8 × 4000 mm, and it is arranged with a downward inclination of 10°. The specification of anchor cable on the lower side of right rib is Φ17.8 × 4500 mm, and it is arranged with a downward inclination of 15°. The spacing and row of anchor cable in two ribs is 1100 × 3200 mm, with pre-tightening force of not less than 120 kN. The roadway support technology and parameters in VIII5-701 panel are shown in Fig. 12.

### Differential pressure relief technology and parameters

**Figure 13:**
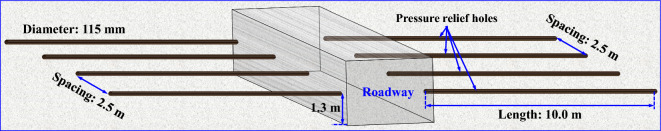
Arrangement of pressure relief holes of roadway affected by residual coal pillar (Zone #A).

In response to the influence of residual concentrated-stress coal pillar in test roadway, it is proposed to adopt differential drilling pressure relief technology in roadway (Zone # A) affected by residual coal pillar. The purpose is to transfer high concentrated stress peak generated by residual coal pillar on roadway to the deeper surrounding rock of two ribs, thereby improving the stress environment of roadway^21^. The technical parameters for drilling pressure relief in two ribs are as follows: For the convenience of construction, the drilling height is 1.30 m from roadway floor, and the drilling diameter is 115 mm. The borehole length is 10.0 m, and the spacing is 2.5 m. The layout of pressure relief holes on both ribs of roadway is shown in Fig. 13.

### Analysis of pressure relief and control effect in coal roadway

#### Displacement of surrounding rock in coal roadway

**Figure 14:**
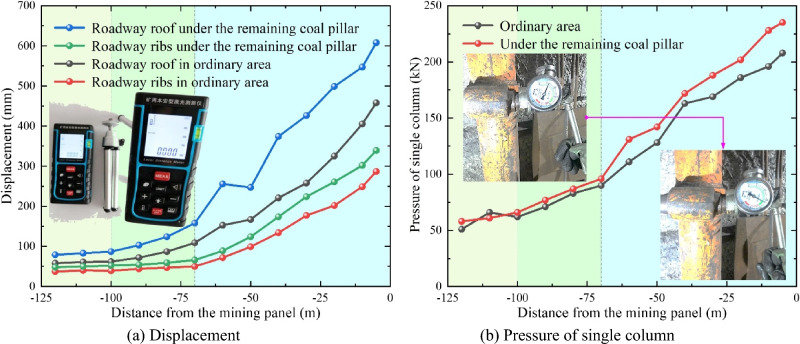
Results curves of mine pressure at different positions of roadway.

In order to verify the application effect of the above regional differential pressure relief and control technology, several measurement stations are set up in coal roadway under the goaf (Zone #B) and residual coal pillar (Zone #A) with the greatest difficulty in surrounding rock control. As shown in Fig. 14a, the deformation of roadway in ordinary zone is significantly smaller than that below residual coal pillar. Overall, the maximum deformation of roadway surrounding rock in any region does not exceed 620 mm. Therefore, after adopting differential pressure relief and control technology, the deformation of roadway is significantly reduced, and the original continuous large-deformation of roadway has been effectively controlled.

#### Pressure of single column

By setting up three monitoring stations for the pressure of single column in ordinary-zone roadway and under residual coal pillar, the results show that pressures of each station in two zones are approximately consistent, and the pressure of single column in roadway under residual coal pillar is significantly greater than that in ordinary zone. The pressure curves of single column of two typical measurement stations in different zones of roadway is shown in Fig. 14b. The pressure in different zones shows significant three-stage characteristics, with the most significant impact being within the range of 70 m from roadway to the panel. Due to intense-mining influence of the panel, the pressure of single column rapidly increases within this range. When the distance between the panel and roadway is reduced to 10 m or even closer, the maximum pressures of single columns of roadway below residual coal pillar and in ordinary zone reach 235 and 208 kN, respectively. In short, the pressure of all single columns is maintained within their reasonable loading range throughout the entire process of the mining panel.

In summary, due to intense-mining influence of VIII5-701 panel, the displacement of roadway and pressure of single column have significantly increased, and exhibit typical three-stage characteristics. Overall, the deformation and pressure of roadway surrounding rock are maintained within a reasonable range. During the service period, there is no significant deformation, failure of anchor cable and single column in test coal roadway. Therefore, adopting the above-mentioned regional, asymmetric, and differential pressure relief and control technology has significantly improved the stress state of roadway surrounding rock, avoided the high cost caused by expansion and repair of roadway, and ensured the safety and stability of roadway in close-distance coal seams. The results provide references for the control of roadway surrounding rock in similar conditions.

## Conclusions

1. The key difficulties in achieving large-deformation control of roadway surrounding rock have been obtained as follows: (1) Deep and complex high-ground-stress. (2) High-concentrated stress of residual coal pillar in close-distance coal seams. (3) Multiple severe-mining disturbance. (4) The roadway roof is irregular and asymmetric goafs. The improvement strategies for controlling roadway surrounding rock have been revealed: (1) Asymmetric reinforcement support for shallow roadway surrounding-rock. (2) Differential pressure relief in roadway under residual coal pillar.
2. It has been determined that the maximum stress peak of roadway surrounding rock under residual coal pillar can reach 38.70 MPa, with a stress concentration coefficient of 1.80, which is 22.90% higher than the peak stress of roadway below the goaf. According to the distribution range of stress field and plasticization failure field in different zones of roadway, the roadway is divided into two key zones along the axial direction, namely, ordinary zone and disturbance zone affected by residual coal pillar. The disturbance distance of roadway under residual coal pillar is determined to be 44.60 m.
3. The evolution laws of plastic zone in roadway during the mining process is explored, revealing the differential, asymmetric, and non-uniform failure patterns of roadway under irregular goafs and residual coal pillar. It clarifies the contour lines of deviatoric stress and vertical stress of roadway surrounding rock in close-distance coal seams, and determines the support strategy of asymmetric inclined arrangement of anchor cables and passing through its boundary lines.
4. The asymmetric combined support of inclined anchor cables and W-steel-strip in ordinary roadway, and differential joint control technology of combined support and drilling pressure relief in roadway affected by residual coal pillar have been proposed. The on-site mine pressure results indicate that large deformation of roadway under irregular goafs and residual coal pillar in deep close-distance coal seams has been effectively controlled, and the stress environment has been significantly improved. The results provide technical references for the control of roadway surrounding rock in similar deep and complex conditions.

## Data Availability

All data and models or used during the study appear in the submitted article.
